# What underlies emotion regulation abilities? An innovative programme based on an integrative developmental approach to improve emotional competencies: Promising results in children with Prader–Willi syndrome

**DOI:** 10.3389/fpsyt.2022.1038223

**Published:** 2022-12-21

**Authors:** Nawelle Famelart, Gwenaelle Diene, Sophie Çabal-Berthoumieu, Mélanie Glattard, Catherine Molinas, Maithe Tauber, Michèle Guidetti

**Affiliations:** ^1^Laboratory CLLE, University of Toulouse, CNRS, Toulouse, France; ^2^Centre de Référence du Syndrome de Prader-Willi, CHU Toulouse, Toulouse, France; ^3^Laboratory CPTP, University of Toulouse, CNRS, INSERM, Toulouse, France

**Keywords:** emotion competencies, developmental model, Prader–Willi syndrome, training programme, integrative approach, children

## Abstract

**Background:**

This study aimed to test the effect of a new training programme on emotional competencies, named EMO-T, and to show the value of an integrative developmental approach. This approach postulates that the emotion regulation disturbances commonly observed in neurodevelopmental disorders are the consequence of potential disruptions in the prerequisite emotion skills. This integrative approach is particularly suitable in the case of complex and multidimensional disorders such as Prader–Willi syndrome (PWS), a rare genetic disease.

**Methods:**

We examined the emotion expression, recognition, comprehension, and regulation skills in 25 PWS children aged 5–10 and 50 typically developing children (TD) aged 3–10. After a pre-test session, half of the PWS children participated in the EMO-T programme with their regular therapist for 6 weeks, while the other half continued their usual rehabilitation programme. Two post-test sessions were conducted, one at the end of the programme and one 3 months later.

**Results:**

At pre-test, PWS children displayed a deficit in the four emotional competencies (EC). PWS children who participated in the EMO-T programme showed a significant and sustainable post-test improvement regarding voluntary expression and emotion recognition abilities, such that the level reached was no longer different from the baseline level of TD children. They also tended to improve in their emotion regulation, although they received no specific training in this skill.

**Discussion:**

These results support that emotion regulation abilities require prerequisite emotion skills, which should be more fully considered in current training programmes. Because emotion regulation disorders strongly impact all areas of life, an integrative developmental approach appears crucial especially in the case of neurodevelopmental disorders. Further studies should be conducted to explore this perspective.

## Introduction

### A developmental model of emotion competencies

In the last two decades, there has been growing interest in the study of emotional competencies (EC) for their key role in social adaptation. EC refer to the ability to use emotions daily, including their expression, recognition, comprehension, and regulation (1). Most studies tend to focus on these various aspects of EC independently, without considering what underlies them and how they interact during development. Disturbances in these EC are commonly observed in neurodevelopmental disorders. Notably, emotion regulation disturbances can strongly impact daily life (2). To improve the effectiveness of assessment tools and training programmes, it appears important to adopt an integrative developmental approach, i.e., an approach that tackles all areas of emotional competencies, considering their development timeline, and including the child’s various caregivers.

From a developmental perspective, the different EC emerge in a hierarchical manner across childhood and adolescence (3–5). Expression and recognition abilities, which are the pillars on which interpersonal relationships are established, emerge very early during the first months of life (6). They can be considered as the basis of the development of emotion comprehension (7). This skill refers to comprehension of the causes and consequences of emotions according to the context and emerges later, at the end of the first year (8). Through the expression, recognition and comprehension skills, the individual gradually conceives of emotion as a concept (9). The construction of this “emotion theorizing” is closely linked to the emergence of the theory of mind for which a pivotal phase is typically observed from the age of 4. “Emotion theorizing” also appears necessary for the regulation of emotions and, by extension, adaptation in general (7, 8, 10–12). Emotion regulation skills develop more steadily across childhood and adolescence (4). In the early years, emotion is mostly co-regulated by parents or caregivers. Interpreting their child’s expressions, parents implement strategies to help regulate them (e.g., trying to distract the child’s attention to stop them crying). Over the course of development, a child broadens its repertoire, regulating emotions by increasingly sophisticated strategies. At first, this goes through a co-regulating process, but it also involves learning how to recognize and to emotionally master daily life situations. At the end of the second year, children begin to mobilize some regulation strategies on their own initiative (13–16). They are gradually able to manage their reactions through improved voluntary control of expression, an ability that starts to be particularly efficient from the age of 4 (17–20).

Given this developmental model, emotion regulation abilities seem to be strongly dependent on the expression, recognition, and comprehension skills, which can be considered as prerequisites (9). In this perspective, emotion regulation disorders–the most common reason for complaints by the entourage (2, 21)—might be the consequence of potential disruptions in the prerequisite skills. Thus, there is much to gain by basing EC training programmes on this developmental perspective.

### Training programmes of emotion competencies

To date, various intervention programmes have been developed to improve EC in children. For the most part, they were developed to prevent (e.g., school context, at-risk populations) or to remedy (e.g., populations with neurodevelopmental disorders) behavioral and social adjustment problems.

Integrative programmes such as the *Social-Emotional Intervention* (13), the *Preschool PATHS* (22), the *SMILE* (23) or the *RULER* (24) programme showed the value of including parents or teachers in the promotion of emotion abilities in children. The *Emotion-Based Prevention* programme (25), which included school and family, showed an improvement in the emotion knowledge and the regulation abilities in children after training in the identification and comprehension of emotions. The study also revealed that the level of emotion knowledge moderated the effect of the programme on both regulation abilities and on social skills. These results once again reinforce the developmental model of EC. The main challenge of training programmes is to promote the transfer of skills acquired during the training sessions into all areas of daily life. The integration of most of the child’s caregivers (i.e., family, teachers, therapists) into the programme appears to be crucial, as highlighted in Hadwin, Howlin, and Baron–Cohen’s programme (26).

Immersive programmes also showed their ability to remediate emotional difficulties. Computer programmes such as *Emotion Trainer* (27), *Mind Reading* (28), or *FaceSay* (29) were specifically developed to train emotion recognition and comprehension abilities in children with Autistic Spectrum Disorders (ASD). The main advantage of the computer format is the possibility of regular use at home. These training programmes are progressive and game-based, which adds an ecological value to the tool. Indeed, the game context (especially board games) seems to be relevant to promote the development of emotion regulation competencies in young children (30).

However, a developmental approach may be lacking in many of these programmes. Several of them focus only on one specific competence, and most of them do not include voluntary expressive skills, which are key for emotion regulation and social adjustment abilities. As a few studies have shown, it is advantageous to consider all the prerequisite skills required to develop a competence. For instance, the programme developed by Begeer et al. (31) aimed to improve social cognition in children with ASD, including therapists and families. The protocol started by a reinforcement of competencies considered as precursors of theory of mind (including imitation, perception, interactions with others). In the following steps, sessions focused on training the comprehension of social situations, the thoughts of others, and lastly the ability to reason from another person’s perspective. Results showed a significant improvement in basic skills and the conceptual comprehension of theory of mind. The authors also observed a lack of transfer of the skills acquired into daily life, however, due to the generalization difficulties of individuals with ASD.

As mentioned in the review by Mazefsky et al. (21), a holistic approach to EC is crucial in the case of neurodevelopmental disorders. Emotion regulation difficulties should rather be considered as a symptom because they are part of a broader, multidimensional problem. An integrative programme based on a developmental model appeared particularly suitable for complex neurodevelopmental disorders such as Prader–Willi syndrome.

### The case of Prader–Willi syndrome

Prader–Willi syndrome (PWS) is a rare genetic disease related to the loss of expression of paternally-inherited genes on chromosome 15 in the region q11–13. PWS is a complex neurodevelopmental disorder characterized by a significant dysfunction of the endocrine system, leading to neonatal hypotonia, growth retardation, eating disorders, and sleep disturbances (32). The phenotype also comprises learning difficulties and many psychological dysfunctions. People with PWS display a mild or moderate intellectual disability (average IQ of 60–70), memory, executive, and perceptive dysfunctions (33–35). They exhibit a language and a motor delay (36–38). In terms of social abilities, people with PWS show social maladjustment and many behavioral disorders, with several autistic features (39, 40). The literature describes a symptomatology such as tantrums, emotional lability, impulsive behavior, lack of empathy and of emotional regulation, anxiety and difficulties of social adaptation (35, 37, 40, 41), suggesting disturbances in social and emotional competencies. These problems strongly impact daily life, constituting the main reason besides hyperphagia for the family burden (42).

Regarding EC, the literature reports difficulties in the recognition and comprehension of basic emotions. Individuals with PWS make on average 10–20% more errors in identifying and assigning emotions than the typical population, even when matched for developmental age (35, 43). They take very little information into account to judge a situation, with difficulties accessing a global representation, focusing instead on details that are mostly irrelevant (39, 43, 44). The same difficulties are observed in face and voice processing (45, 46). This particularity is likely to compromise their capacity for emotional recognition and thus to place them at a disadvantage in everyday situations. In addition, the emotional expressions of PWS children are particularly poor and equivocal, making it difficult to interpret them (9).

Very few intervention programs aimed at directly improving EC in the PWS population exist, however. Most of the studies focus on hormonal treatments [Ghrelin, Oxytocin (47, 48)] or medical protocols [vagus nerve stimulation—tVNS (49)], showing some beneficial effects on social and emotional skills. Recently, two studies reported promising results on two online intervention programs aimed at developing social skills. The BOSS program (50) was offered to adolescents and young adults with PWS. The sessions were collective and took place by videoconference, 3 times a week for 10 weeks. The results showed an interesting effect of the program on socialization (friendly relations and quality of interactions with peers). The PRETEND (42) program was aimed at 3–5 year-old children with PWS and the play skills intervention work was done through the parents. The online coaching sessions took place weekly for 8 weeks. Again, the results were promising, showing interesting improvements in cognitive and affective play skills for some of the children with PWS.

To date, however, no intervention program specifically addressing EC in children with PWS has been tested, despite the need. Considering the complexity and the multidimensional disorders related to PWS, the extent of the EC difficulties involved, and their consequences on daily life, the aim of the present study was to test the effect of a training programme on EC, named *EMO-T*. We focused on a relatively narrow age range, which is rare in studies of PWS. The period of school age is typically a pivotal phase in emotional development. During this period, the process of “emotion theorizing” is strongly accentuated in the light of the development of other skill areas [cognitive, socialization, learning, autonomy; (3, 7)]. We postulated that a programme based on an integrative developmental approach would enable children to make significant and lasting progress in EC.

## Materials and methods

### Population

The study population was composed of 25 children with PWS aged 5;5–10;5 years. The average IQ was 75.7 and the average intellectual developmental age was 5;7 years. The children were divided into two groups, one experimental group (PWS-EG) and one control group (PWS-CG) (see Table 1 for details). The distribution was quasi-randomized. It was carried out in such a way that at any time of inclusion (spanned 18 months), the two groups were comparable in terms of sex ratio, chronological age, and IQ. Group comparison analysis (Student *t*-test) showed that the two groups had equivalent IQ, but showed a slight difference—albeit not significant—in term of age (see Table 1). The parents were not informed which group their child was assigned to. The families socioeconomic status (SES) was measured by the Hollingshead scale (51), which is based on marital status, education level and current occupation. SES levels range between 1 and 5, 1 corresponding to a low SES and 5 to a high one. The families in the PWS sample had a median of 4 (middle-high SES). There was no significant difference between the PWS-EG and PWS-CG groups (see Table 1).

**TABLE 1:** Descriptive characteristics for PWS and TD groups.

	All PWS	PWS-EG	PWS-CG	PWS groups equivalence	TD-CA	TD-DA
N	25	13	12	–	25	25
(girls/boys)	(14/11)	(7/6)	(7/5)		(14/11)	(14/11)
**CA**						
Mean (SD)	7;6 (1;6)	8;0 (1;8)	7;0 (1;1)	*t*_(23)_ = −1.754; *ns*; *d* = *0.73*	7;6 (1;5)	5;7 (1;4)
Range	5;5–10;4	5;9–10;4	5;5–8;7	(Student *t*-test)	5;2–10;10	3;0–8;4
**IQ**						
Mean (SD)	75.7 (17.1)	75.9 (19.6)	75.4 (14.8)	*t*_(23)_ = −0.072; *ns*; *d* = *0.03*		
Range	44–103	44–103	50–94	(Student *t*-test)		
**DA**						
Mean (SD)	5;7 (1;5)	5;11 (1;7)	5;2 (1;1)	*t*_(23)_ = −1.273; *ns*; *d* = *0.53*		
Range	3;2–9;2	4;3–9;1	3;2–6;9	(Student *t*-test)		
**SES**						
Median	4	4	3.5	*W* = 60*; ns*	4	5
(Q1–Q3)	(3–4)	(4–4)	(3–4)	(Wilcoxon test)	(3–5)	(4–5)

Means, ranges, and SDs of chronological age (CA), intellectual developmental age (DA), and full-scale IQ (IQ); Medians, 1st and 3rd quartiles of socioeconomic status (SES); Results of PWS groups equivalence tests. *ns*, not significant; age, [years; months]; PWS-EG, PWS experimental group; PWS-CG, PWS control group; TD-CA, TD group with chronological age matched; TD-DA, TD group with development age matched.

Fifty children with typical development (TD) also participated in the study, divided into two groups (see Table 1). The first group consisted of 25 children matched to PWS children by sex and chronological age (TD-CA group). The second group consisted of 25 other children matched to PWS children by sex and intellectual developmental age (TD-DA group). Group comparison analysis (Student t-test) confirmed that the mean age of the TD-CA group was equivalent to the mean chronological age of the PWS group [*t*_(48)_ = 0.072; *ns*; *d* = 0.020]. The mean age of the TD-DA was similar to the mean developmental age of the PWS group [*t*_(48)_ = −0.025; *ns*; *d* = 0.007]. None of the TD children had any academic or learning delays. The SES levels of families in the TD-DA group were higher (median = 5) than the TD-CA and PWS groups (median = 4) [Kruskal–Wallis test: χ^2^_(2, *N* = 75)_ = 7.898, *p* = 0.019; see Table 1]. However, this difference cannot explain the results obtained in experimental tasks, which varied in the other direction (see Section “Results”).

**FIGURE 1:**
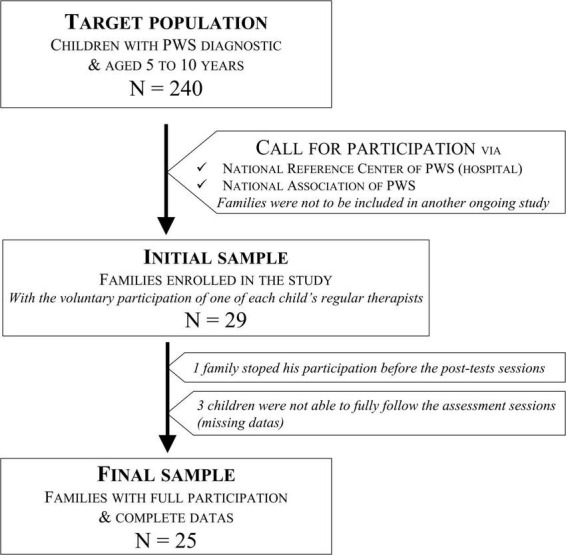
Prader–Willi syndrome (PWS) participants flow chart.

A power analysis was conducted on emotion regulation rate. The estimation of the delta and the standard deviation was based on previous studies with individuals suffering from neurodevelopmental disorders on the one hand, and with typically developing individuals on the other hand. Setting the alpha at 0.05 and power at 0.80 yielded a total sample size of 32 (16 for each group). The recruitment of PWS children was carried out through the PWS French National Reference Centre and the French National Association of PWS. Initially, 29 families were enrolled in the study, corresponding to 12% of the target population. One family stopped its participation before the end of the protocol. Three children were not able to fully follow the assessment sessions and were removed from the analysis because of missing data. The participation of PWS families in the EMO-T programme was established in collaboration with one of each child’s therapists (speech therapist, psychomotor therapist, or psychologist). The programme was included in the child’s regular care programme (see the flowchart in Figure 1).

### Materials

Given our objectives, the challenge was two-fold. The first task was to assess the children with respect to four emotional competencies: expression, recognition, comprehension, and regulation. The assessment tasks used were created or adapted from previous studies. The second challenge was to develop a training programme consisting of exercises aiming to improve emotional competencies. As the developmental model considers expression, recognition and comprehension as prerequisite skills for emotion regulation, the exercises of the EMO-T programme focused exclusively on these three competencies. The exercises used were created or adapted from previous studies in order to maintain consistency with the assessment tasks, without being identical.

The tasks and exercises are presented briefly below (for more details, see Supplementary Appendix A).

#### Assessment tasks

##### Expression

The two tasks used were created to assess spontaneous emotional reactions and voluntary production of emotional expressions [from Famelart et al.’s (9) study]. Scores are expressed as a proportion between 0 and 1, 1 meaning that the emotion is fully detectable and matches the theoretical pattern. The *EMOrea Task* consisted in recording the facial reactions of the child while he/she was watching a funny video clip (that was likely to induce the emotion of joy in children). The *EMOmim Task* consisted in asking the child to produce emotional facial expressions (voluntary expressions) of joy, sadness, fear, and anger.

##### Recognition

Three tasks with different levels of complexity were used to assess the ability to recognize and name the emotions of joy, sadness, fear, and anger. Scores are presented in percentage of correct responses. In the *Identification task*, the participant had to point out the picture of the person expressing the emotion specified in the verbal instruction. In the *Matching task*, the participant had to select the picture of the person expressing the same emotion as in the target picture presented at the top of the screen. In the *Naming task*, the participant had to say which emotion the person was feeling in a short video.

##### Comprehension

The *AJQ task* (Affective Judgment Questionnaire) was used to assess the ability to attribute an emotion according to the context of stories. The AJQ task consisted in presenting short illustrated stories and asking the child to say which emotion the character felt (emotions targeted: joy, sadness, fear, and anger) and to justify their response. Scores were based on emotion attribution and kind of justifications, and they are presented as the percentage of correct responses.

##### Regulation

To assess the emotion regulation skills, we used the *Emotion Regulation Checklist* (ERC—French version), which is a questionnaire completed by parents. Scores of the composite scale range between 0 and 4, and the higher the score, the better the regulation abilities.

#### EMO-T programme

The EMO-T programme was conducted by one of the child’s therapists (speech therapist, psychomotor therapist, or psychologist) and was included in the child’s regular care programme. The therapist received a half-day training session on the programme proposed by the researchers. The programme was applied weekly over six 30-min sessions. Each session consisted of the same exercises; however, the supports and stimuli evolved so that the task became more complex as the sessions progressed.

Each session included exercises to train the abilities of expression, recognition, and comprehension of emotions. All the exercises were based on the child’s justifications and arguments and the discussion with the therapist. The sessions followed the same sequence of five exercises:

(1) Sorting of static emotional facial expressions–*Recognition*;
(2) Naming of dynamic emotional expressions (vocal and facial) –*Recognition*;
(3) Mimicking emotions and recognizing them–*Expression*;
(4) Emotion attribution–*Comprehension*;
(5) Narration of emotional experience–*Comprehension*.

### Experimental design

For each test session, the children were individually interviewed in a quiet room at home. All the children were met by the same experimenter, a researcher trained as a psychologist. The tasks were always presented in the same order: EMOrea, EMOmim, Matching, Identification, Naming, and AJQ. During this phase, the parents completed the ERC questionnaire.

All the children–PWS and TD children–were first assessed in a pre-test session. The PWS children included in the experimental group (PWS-EG) then received the training programme conducted by the previously trained therapist (see above), over 6 weeks (weekly 30-min sessions). During this phase, PWS children from the control group (PWS-CG) continued their usual rehabilitation with their therapist. In the third step, all the PWS children were retested, immediately after the end of the programme (post-test 1) and again 3 months later (post-test 2). Children in the PWS-CG group followed the training programme at the end of the protocol and their therapist was trained only at this time.

## Results

### Design of analyses

To test the efficiency of the EMO-T programme on the children’s EC, analyses were conducted in three steps for each task, considering the PWS Experimental Group as a reference for analyses: (1) comparison of PWS groups with the TD groups at the pre-test session (group effect); (2) comparison of the trajectory evolution of the two PWS groups between the pre- and the two post-test sessions (interaction effect); (3) comparison of the PWS groups at the second post-test session with the TD groups considered as a baseline (from the pre-test session). We used a Generalised Linear Mixed Model (GLMM) to adapt the analytical model to the specificity of the variable distribution and to integrate random effects (repeated measures). Preliminary analyses showed that age, IQ, and sex had no significant interaction or influence on any of the main effect shown.

### Analysis results

**FIGURE 2:**
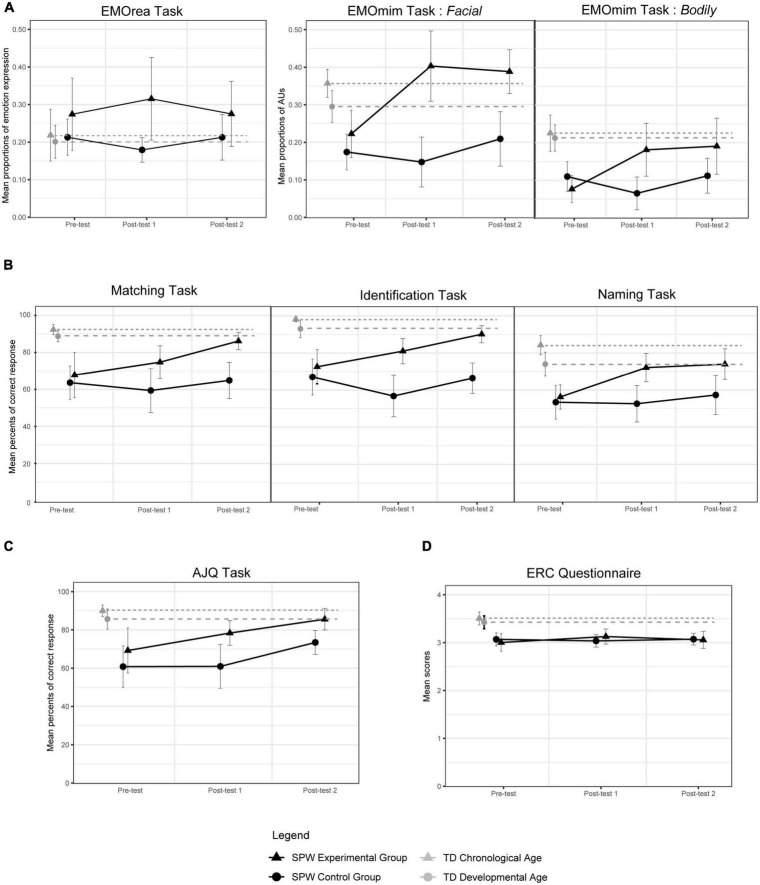
Graphical results of the three test sessions for each task. **(A)** Expression. **(B)** Recognition. **(C)** Comprehension. **(D)** Regulation.

Figure 2 and Table 2 summarize the results and statistical analyses for each task. All the details are presented in the Supplementary Appendix B.

**TABLE 2:** Summary of results of the three steps of analyses for each task.

Task	Base level	Sign improvement		Level reached
			
	Comparison group pre-test	EG: Pre≠post1	EG: Post1≠2	Comparison group post-test 2
**Expression**				
EMOrea	**EG**–CG–DA–CA	→	→	**EG**–CG–DA–CA
	*ns*	*Ns*		*ns*
EMOmim				
*Facial*	**EG**–CG < DA–CA	↗°	→°	CG < DA–CA–**EG**
*Bodily*	**EG**–CG < DA–CA	↗°	→	**EG**–CG < DA–CA
	***	***		***
**Recognition**				
Matching	**EG**–CG < DA < CA	↗°	↗°	CG < DA–**EG** < CA
Identification	**EG**–CG < DA < CA	↗°	↗°	CG < DA–**EG** < CA
Naming	**EG**–CG < DA < CA	↗°	→°	CG < DA–**EG** < CA
	***	***		***
**Comprehension**				
AJQ	CG <**EG** < DA–CA	(↗°)	(↗°)	CG < DA–CA–**EG**
	***	*Ns*		***
**Regulation**				
ERC	**EG**–CG < DA–CA	(↗°)	(→)	**EG**–CG < DA–CA
	***	¤		***

EG, PWS experimental group; CG, PWS control group; CA, TD group with chronological age matched; DA, TD group with development age matched. Comparisons group (X-Y: no difference score between X and Y; X < Y: X’s score is lower than Y’s score; X ≤ Y: X’s score tends to be lower than Y’s score). The improvement sign shows the difference between pre-test and post-test 1/between post-test 1 and 2 for the PWS-EG group (→: no difference; ↗: increase; °: higher score than PWS-CG group). *P*-values (*ns*: not significant; ***p < 0.001; ¤: tendency 0.05 < *p* < 0.08). *P*-values refer to group effect analyses (comparison group at pre-test and at post-test 2) and to interaction effect analyses (two PWS groups × three test sessions).

Regarding the first step of analyses, results from the pre-test session indicated a strong group effect for all tasks, except for the EMOrea task. The scores of the two PWS groups were significantly lower than those of the two TD groups. In addition, analyses showed that the scores of the two PWS groups were statistically similar. However, for the AJQ task, the scores of the PWS-EG group tended to be higher than the scores of the PWS-CG group.

In the second step, results revealed a strong interaction between the PWS groups and the test sessions for the EMOmim task and the three recognition tasks. The scores of the PWS-EG group increased significantly between the pre-test session and the first post-test session, in which they achieved significantly higher scores than the PWS-CG group. The scores of the PWS-EG group between the two post-test sessions remained statistically unchanged in the EMOmim task and the Naming task, while the group showed a significant increase in the Matching and Identification tasks.

No significant interaction was observed for the EMOmim task and the AJQ task. Nevertheless, results from the AJQ task indicated a group effect, with the PWS-EG group mainly displaying higher scores than the PWS-CG group. Results also highlighted an effect of the test session independently of the group. Specifically in the PWS-EG group, analyses showed a significant increase in scores between the pre-test session and the first post-test session, and a strong tendency to increase between the first and the second post-test session.

In the ERC questionnaire, analyses showed a strong tendency toward interaction. Scores in the PWS-EG group increased between the pre-test session and the first post-test session, without any significant differences between the two post-test sessions.

Lastly, in the third step of the analyses, we observed a significant group effect for the EMOmim task (Facial modality only), the three recognition tasks and the AJQ tasks. Scores in the PWS-EG group were significantly higher than those in the PWS-CG group at the second post-test session. The scores of the PWS-EG group no longer statistically differed from those of the two TD groups in the EMOmim task (Facial modality only) and in the AJQ task. In the three recognition tasks, the scores of the PWS-EG group no longer statistically differed from those of the TD-DA group, but they remained significantly lower than those of the TD-CA group. In the ERC questionnaire, scores in the PWS-EG group remained significantly lower than those of the two TD groups and statistically similar to those of the PWS-CG group. In the EMOrea task, the four groups remained statistically similar.

## Discussion

This study aimed to test the effect of the EMO-T programme on EC, based on an integrative developmental approach. The programme was applied to children with PWS as they display specific patterns of emotion regulation which are part of a broader and multidimensional problem.

Analyses showed four main results. First, PWS children displayed a specific deficit in the four EC (i.e., expression, recognition, comprehension, and regulation of emotions). Second, at post-test 2, PWS children who benefited from the EMO-T programme showed a significant improvement in their voluntary expression and recognition abilities. Third, this level reached was maintained over time, and even continued to increase beyond the programme (recognition). Fourth, the post-programme level of PWS children was no longer different from the baseline level of TD children matched by intellectual developmental age, nor even of TD children matched by chronological age (voluntary expression and comprehension).

Lastly, PWS children in the experimental group tended to improve in their emotion regulation, although they received no specific training in this skill. These results support the developmental model of EC, showing that emotion regulation abilities require expression, recognition, and comprehension skills. Such prerequisite skills should be more fully considered in current training programmes.

Nevertheless, the improvement in emotion regulation was limited and was not sustained over time, which can be attributed to several reasons. First and foremost, the mixed results regarding emotion comprehension could be a cause of this. Since comprehension abilities require several skills in many areas, especially cognitive skills, they are considered as the pillar of emotional development (7, 8, 11). In this light, the programme should include the remediation of cognitive and executive abilities, which are also heavily involved in EC, as highlighted in the study by Li et al. (52) or the study by Weiss et al. (53).

The limited sample size could also have made the results less consistent. The fact that the theoretical sample determined from the power analysis could not be reached may also explain the trend results in the emotion regulation scale. In addition, the great variability of profiles existing in PWS (35) made it more complex to set up suitable tasks. This can be illustrated by the three children who were withdrawn from the analyses because they showed too low a level of understanding and were not able to follow the instructions.

The unsustainable improvement in emotion regulation could also be due to the programme format. The EMO-T programme was applied over 6 weeks, a choice that was determined by organizational constraints, but this can be considered as a minimum duration. From the other programmes, a longer period of 3 months seems to offer a better opportunity of stabilizing the progression (25, 26, 31). Additionally, the EMO-T programme could be made more progressive, focusing initially on the ability to express and recognize emotions, since they are prerequisites for social perception skills (43). From this basis, the sessions could then include exercises on social cognition (emotion comprehension, ToM, etc.). The work on emotion regulation could then be initiated in a final stage. Feedback from therapists supports this proposition, although they also highlighted that a certain degree of redundancy may be relevant for some children who need a routine.

Lastly, the issue of how to measure emotional regulation in children remains unresolved. It is still difficult to develop a standardized methodology to carry out direct observations to measure how and when regulatory strategies are used, and to assess their effectiveness (11). It would be worthwhile further investigating board games in this respect because they create a specific context that requires regulation skills in children (30). The coding system used in Hagstrøm et al.’s (54) study or in Penela et al. (55) also shows interesting possibilities.

To conclude, this study underlined the value of considering a developmental model to promote an EC training programme. This appears crucial especially in the case of neurodevelopmental disorders with multidimensional dysfunctions. The study also showed the importance of adopting an integrative approach, by including the training programme in regular care. The EMO-T programme delivered in a therapeutic context showed an improvement through assessment sessions conducted in the family context. Interestingly, the results on the emotion regulation questionnaire completed by parents showed a potential impact of the programme on daily life. The transfer of acquisitions could be even stronger, however, by considering the “caregiver triad”: family, school, and therapists. Further studies should be conducted to explore this perspective.

## Data availability statement

The raw data supporting the conclusions of this article are available from the corresponding author on reasonable request.

## Ethics statement

Ethical review and approval was not required for the current study in accordance with the local legislation and institutional requirements. Only voluntary children with informed parental consent participated in the study. In line with the latest Declaration of Helsinki (2013), all the children, parents and therapists were fully informed of the nature and characteristics of the study. Written informed consent to participate in this study was provided by the participants’ legal guardian/next of kin.

## Author contributions

NF: elaboration of the study and the method, data collection, data analysis, and writing the manuscript. MT and MGu: study supervision and writing the manuscript. GD, SÇ-B, MGl, and CM: help with the recruitment of participants and the data collection. All authors contributed to the article and approved the submitted version.
