# Supplementary material for: What underlies emotion regulation abilities? An innovative programme based on an integrative developmental approach to improve emotional competencies: Promising results in children with Prader–Willi syndrome
Source: Front Psychiatry. 2022 Dec 21;13:1038223. doi: 10.3389/fpsyt.2022.1038223 (PMC9811587; doi:10.3389/fpsyt.2022.1038223)
Supplement: Supplementary file 1 [file Data_Sheet_1.pdf]

APPENDIX A

Assessment tasks:

Task	Number of items / emotions targeted	Score range	Description	Reference base
**EXPRESSION**				
EMOrea (spontaneous expression)	Funny video clips	0 to 1<br>1 means the emotion is fully detectable	Recording facial reactions of children while they watched a funny video clip (that was likely to induce the emotion of joy in children). Facial reactions were analyzed with FaceReader	FaceReader: *(Noldus, 2016)*<br>Video-clips: *(Famelart & Guidetti, 2017)*
EMOmim (voluntary expression)	4 items<br>Joy, Sadness, Fear & Anger	0 to 1<br>1 means the emotion fully matched with the theoretical pattern	To produce emotional facial expressions (voluntary expressions). Facial and bodily expressions were analyzed by a coding grid based on a combination of FACS and MAX/AFFEX	FACS: *(Ekman & Friesen, 1978)*<br>MAX/AFFEX: *(Izard, 1979; Izard et al., 1983)*
**RECOGNITION**				
Identification *(easiest recognition task)*	32 items<br>Joy, Sadness, Fear & Anger	Percentage of correct responses	To point out the picture (between three options: target, distractor and neutral) of the person expressing the emotion specified in the verbal instruction.	French adaptation of SANAFE *(Lacroix et al., 2009; Reilly & Stiles, 2006)*
Matching	32 items<br>Joy, Sadness, Fear & Anger	Percentage of correct responses	To select the picture (between three options: target, distractor and neutral) of the person expressing the same emotion as in the target picture presented at the top of the screen.	French adaptation of SANAFE *(Lacroix et al., 2009; Reilly & Stiles, 2006)*
Naming *(hardest recognition task)*	16 items<br>Joy, Sadness, Fear & Anger	Percentage of correct responses	To tell what emotion the person was feeling in a short video. In the video, the person produced the emotion using both facial and vocal modalities.	Video clips from the GEMEP Core Set (Bänziger et al., 2012)
**COMPREHENSION**				
AJQ task	8 items<br>Joy, Sadness, Fear & Anger	Percentage of correct responses *(Scores based on attribution and justification)*	To attribute an emotion to the character from a short illustrated story, and to justify the response.	French adaptation of Affective Judgment Questionnaire (Reilly & Delahanty, 1997; Thommen et al., 2010)
**REGULATION**				
ERC	24 items	0 to 4<br>the higher the score, the better the regulation abilities.<br><br>3 scales:<br>- Composite scale<br>- Dysregulation subscale<br>- Regulation subscale	The Emotion Regulation Checklist is a questionnaire completed by parents. ERC aims to assess the emotion regulation skills of the child in daily life.<br>The French version used in this study demonstrates high psychometric qualities (internal consistency alpha varies between .70 and .82, the inter-rate between .86 and .88, the test-retest stability between .90 and .92). ERC is commonly used for children aged 3 to 12 years with typical development, but also for children with a neurodevelopmental disorder or mental delay.	French version of ERC (Nader-Grosbois & Mazzone, 2015; Shields & Cicchetti, 1997)

Program of exercises:

The program was established over **six 30-minute sessions** (weekly sessions).

Each session consisted of the same exercises. However, the supports and stimuli evolved so that the task became **more complex as the sessions progressed**.

All exercises were **based on the child's justifications and arguments** and the discussion with the therapist as both are considered as central in the development of conceptualization. Each exercise aimed to challenge the child **to target the relevant social cues** to assess an emotional situation.

**Competence targeted**	**Material / Stimuli**	**Number of items per session**	**Description**	**Reference base**
**1) Sorting of static emotional facial expressions**				
Recognition *(Categorization)*	Tablet<br>EU Emotion Stimulus Set	8 items: pictures of emotional facial expressions (2 per emotion)<br>Joy, sadness, fear & anger	1/ To sort the images in relation to their valence ("happy" vs. "not happy")<br>2/ To sort specifically the negative emotions (i.e. initially "not happy")	*(O'Reilly et al., 2016)*
**2) Naming of dynamic emotional expressions (vocal and facial)**				
Recognition	Tablet<br>EU Emotion Stimulus Set	4 items: video-clips of emotion expression (facial and vocal)<br>Joy, sadness, fear & anger	To name the emotion expressed by the person in the video and in the soundtrack (presented separately)	*(O'Reilly et al., 2016)*
**3) Mimicking emotions**				
Expression<br>*+ Recognition*	Tablet's front camera	4 items<br>Joy, sadness, fear & anger	1/ To express emotion in front of the camera used as a 'mirror'.<br>2/ To recognize one's own production from shots of the facial expressions.	
**4) Attribution of emotions**				
Comprehension<br>*(Theory of Mind)*	Illustrated scenarii	2 items<br>2 emotions among joy, sadness, fear & anger<br>*(different pairs tested over the six sessions)*	1/ To attribute an emotion to the character from a short illustrated story *(cf. causes of the emotion)*.<br>2/ To select (between three options: target, distractor and neutral) the end of the story that best matched the emotion attributed *(cf. consequences of emotion)*	Adapted from *(Nader-Grosbois & Thirion-Marissiaux, 2011)*
**5) Narration of an emotional experience**				
Comprehension<br>*(Theory of Mind, Metacognition)*		2 items<br>The emotion was related to the story of the previous exercise	To relate a personal situation in which the child felt the same emotion as the one depicted in the previous story.	

REFERENCES

Bänziger, T., Mortillaro, M., & Scherer, K. R. (2012). Introducing the Geneva Multimodal expression corpus for experimental research on emotion perception. *Emotion*, *12*(5), 1161–1179. https://doi.org/10.1037/a0025827

Ekman, P., & Friesen, W. V. (1978). *Manual for the facial action coding system.* Consulting Psychologists Press.

Famelart, N., & Guidetti, M. (2017). The effect of laughter expression modulation on emotional experience in 4 to 10 year-old children. *European Journal of Developmental Psychology*, *14*(3), 311–323. https://doi.org/10.1080/17405629.2016.1201474

Izard, C. E. (1979). *The maximally discriminative facial movement scoring system (MAX).* Instructional Recourses Centre, University of Delaware.

Izard, C. E., Dougherty, L. M., & Hembree, E. A. (1983). *A system for identifying affect expressions by holistic judgments (AFFEX)*.

Lacroix, A., Guidetti, M., Rogé, B., & Reilly, J. (2009). Recognition of emotional and nonemotional facial expressions: A comparison between Williams syndrome and autism. *Research in Developmental Disabilities*, *30*(5), 976–985. https://doi.org/10.1016/j.ridd.2009.02.002

Nader-Grosbois, N., & Mazzone, S. (2015). Validation of the French version of the Emotion Regulation Checklist (ERC-vf). *European Review of Applied Psychology*, *65*(1), 29–41. https://doi.org/10.1016/j.erap.2014.10.002

Nader-Grosbois, N., & Thirion-Marissiaux, A.-F. (2011). Evaluer la compréhension des états mentaux “émotions” et “croyances”. In N. Nader-Grosbois (Ed.), *La théorie de l'esprit: Entre cognition, émotion et adaptation sociale* (pp. 95–124). De Boeck.

Noldus. (2016). *FaceReader: Tool for automatic analysis of facial expression. Reference Manual Version 7.* Noldus Information Technology b.v.

O'Reilly, H., Pigat, D., Fridenson, S., Berggren, S., Tal, S., Golan, O., Bölte, S., Baron-Cohen, S., & Lundqvist, D. (2016). The EU-Emotion Stimulus Set: A validation study. *Behavior Research Methods*, *48*(2), 567–576. https://doi.org/10.3758/s13428-015-0601-4

Reilly, J., & Delahanty, M. (1997). *Affective Judgment Questionnaire.* SDSU.

Reilly, J., & Stiles, J. (2006). *SANAFE*.

Shields, A., & Cicchetti, D. (1997). Emotion regulation among school-age children: The development and validation of a new criterion Q-sort scale. *Developmental Psychology*, *33*(6), 906–916. https://doi.org/10.1037/0012-1649.33.6.906

Thommen, E., Suárez, M., Guidetti, M., Guidoux, A., Rogé, B., & Reilly, J. S. (2010). Comprendre les émotions chez les enfants atteints d'autisme: Regards croisés selon les tâches. *Enfance*, *2010*(03), 319–337. https://doi.org/10.4074/S0013754510003083
