# Supplementary material for: What underlies emotion regulation abilities? An innovative programme based on an integrative developmental approach to improve emotional competencies: Promising results in children with Prader–Willi syndrome
Source: Front Psychiatry. 2022 Dec 21;13:1038223. doi: 10.3389/fpsyt.2022.1038223 (PMC9811587; doi:10.3389/fpsyt.2022.1038223)
Supplement: Supplementary file 2 [file Data_Sheet_2.pdf]

## APPENDIX B

To test the efficiency of the EMOT programme on the children's EC, analyses were conducted in three steps for each task, considering the PWS Experimental Group as a reference for analyses: 1) comparison of PWS groups with the TD groups at the pre-test session (group effect); 2) comparison of the trajectory evolution of the two PWS groups between the pre- and the two post-test sessions (interaction effect); 3) comparison of the PWS groups at the second post-test session with the TD groups considered as a baseline (from the pre-test session).

<u>General legend</u>:

- GLM group and interaction effect results: Wald Chi-Square test
- GLM pairwise comparison results: Wald test *z.*

### EXPRESSION

**Table 1- EMOrea Task:** Mean (SD) and GLM results for each group and test session.

| Group | Pre-test | | Post-test 1 | | Post-test 2 | |
|---|---|---|---|---|---|---|
| GLM Bêta Dist. | Group effect *ns* | | Interaction effect *ns* | | Group effect *ns* | |
| PWS-EG | 0.274 *(0.177)* | Pre-Post1 comp. *ns* | 0.315 *(0.202)* | Post1-2 comp. *ns* | 0.275 *(0.159)* | |
| PWS-CG | 0.274 *(0.177)* | PWS-EG comp. *ns* | 0.179 *(0.058)* | PWS-EG comp. *ns* | 0.213 *(0.107)* | PWS-EG comp. *ns* |
| TD-DA | 0.213 *(0.085)* | *ns* | - | - | - | *ns* |
| TD-CA | 0.201 *(0.112)* | *ns* | - | - | - | *ns* |

**Table 2- EMOmim Task:** Mean (SD) and GLM results for each group and test session

| Group | Pre-test | | Post-test 1 | | Post-test 2 | |
|---|---|---|---|---|---|---|
| GLM Bêta Dist. | Group effect $\chi^2_{(3, N=74)}$= 42.707, *p*< .001 | | Interaction effect $\chi^2_{(2, N=25)}$=23;577, *p*< .001 | | Group effect $\chi^2_{(3, N=74)}$=23;945, *p*< .001 | |
| **FACIAL** | | | | | | |
| PWS-EG | 0.223 *(0.116)* | Pre-Post1 comp. *z*=-3.649, *p*<.001 | 0.403 *(0.165)* | Post1-2 comp. *ns* | 0.388 *(0.108)* | |
| PWS-CG | 0.174 *(0.084)* | PWS-EG comp. *ns* | 0.148 *(0.117)* | PWS-EG comp. *z*=-3.095, *p*=.002 | 0.209 *(0.128)* | PWS-EG comp. *z*=-3.062, *p*=.002 |
| TD-DA | 0.295 *(0.106)* | *z*=2.418, *p*=.016 | - | - | - | *ns* |
| TD-CA | 0.357 *(0.095)* | *z*=5.879, *p*<.001 | - | - | - | *ns* |
| **BODILY** | | | | | | |
| PWS-EG | 0.076 *(0.067)* | Pre-Post1 comp. *z*=-4.459, *p*<.001 | 0.181 *(0.124)* | Post1-2 comp. *ns* | 0.191 *(0.137)* | |
| PWS-CG | 0.110 *(0.070)* | PWS-EG comp. *ns* | 0.065 *(0.077)* | PWS-EG comp. *z*=-4.153, *p*<.001 | 0.112 *(0.082)* | PWS-EG comp. *ns* |
| TD-DA | 0.213 *(0.087)* | *z*=5.649, *p*<.001 | - | - | - | *z*=3.052, *p*=.002 |
| TD-CA | 0.225 *(0.123)* | *z*=5;879, *p*<.001 | - | - | - | *z*=3.235, *p*=.001 |

**RECOGNITION**

**Table 3- Matching, Identification and Naming Task:** Mean (SD) and GLM results for each group and test session.

| Group | Pre-test | | Post-test 1 | | Post-test 2 | |
|---|---|---|---|---|---|---|
| GLM Binom. Dist. | Group effect $\chi^2_{(3, N=73)}$= 175.398, *p*< .001 | | Interaction effect $\chi^2_{(2, N=25)}$=38.578, *p*< .001 | | Group effect $\chi^2_{(3, N=73)}$=131.905, *p*< .001 | |
| **MATCHING** | | | | | | |
| PWS-EG | 67.79 *(22.58)* | Pre-Post1 comp. *z*=-2.319, *p*=.020 | 74.76 *(16.36)* | Post1-2 comp. *z*= 3.560, *p*<.001 | 86.20 *(8.26)* | |
| PWS-CG | 63.64 *(15.33)* | PWS-EG comp. *ns* | 59.38 *(21.07)* | PWS-EG comp. *z*=-2.583, *p*=.010 | 64.84 *(17.38)* | PWS-EG comp. *z*=-3.620, *p*<.001 |
| TD-DA | 88.80 *(7.37)* | *z*=4.358, *p*<.001 | - | - | - | *ns* |
| TD-CA | 92.38 *(6.93)* | *z*=5.724, *p*<.001 | - | - | - | *z*=2.194, *p*=.028 |
| **IDENTIFICATION** | | | | | | |
| PWS-EG | 72.60 *(16.98)* | Pre-Post1 comp. *z*=-2.978, *p*=.003 | 81.01 *(12.53)* | Post1-2 comp. *z*= 2.934, *p*=.003 | 90.10 *(8.09)* | |
| PWS-CG | 67.05 *(16.44)* | PWS-EG comp. *ns* | 56.77 *(19.80)* | PWS-EG comp. *z*=-4.016, *p*<.001 | 66.41 *(14.48)* | PWS-EG comp. *z*=-4.417, *p*<.001 |
| TD-DA | 93.00 *(11.91)* | *z*=5.700, *p*<.001 | - | - | - | *ns* |
| TD-CA | 98.12 *(3.38)* | *z*=7.902, *p*<.001 | - | - | - | *z*=4.392, *p*<.001 |
| **NAMING** | | | | | | |
| PWS-EG | 56.25 *(11.97)* | Pre-Post1 comp. *z*=-3.432, *p*=.006 | 72.12 *(14.12)* | Post1-2 comp. *ns* | 73.96 *(14.56)* | |
| PWS-CG | 53.41 *(15.40)* | PWS-EG comp. *ns* | 52.60 *(17.37)* | PWS-EG comp. *z*=-2.604, *p*=.009 | 57.29 *(18.62)* | PWS-EG comp. *z*=-2.150, *p*=.031 |
| TD-DA | 73.96 *(16.14)* | *z*=2.762, *p*=.006 | - | - | - | *ns* |
| TD-CA | 84.25 *(13.27)* | *z*=4.779, *p*<.001 | - | - | - | *z*=2.102, *p*=.036 |

**COMPREHENSION**

**Table 4- AJQ Task:** Mean (SD) and GLM results for each group and test session.

| Group | Pre-test | | Post-test 1 | | Post-test 2 | |
|---|---|---|---|---|---|---|
| GLM Binom. Dist. | Group effect $\chi^2_{(3, N=73)}$= 83.915, *p*< .001 | | Interaction effect *ns* | | Group effect $\chi^2_{(3, N=73)}$=42.507, *p*< .001 | |
| PWS-EG | 85.58 *(10.33)* | Pre-Post1 comp. *z*=-2.157, *p*=.031 | 78.37 *(11.86)* | Post1-2 comp. *z*=-1.935, *p*=.053 | 85.58 *(10.33)* | |
| PWS-CG | 60.80 *(18.35)* | PWS-EG comp. *z*=-1.728, *p*=.084 | 60.94 *(20.32)* | PWS-EG comp. *z*=-2.862, *p*=.042 | 73.44 *(11.03)* | PWS-EG comp. *z*= -3.94, *p*<.001 |
| TD-DA | 85.68 *(13.09)* | *z*= 4.673, *p*<.001 | - | - | - | *ns* |
| TD-CA | 90.00 *(7.65)* | *z*=6.178, *p*<.001 | - | - | - | *ns* |

No significant interaction was observed between the PWS group and the test session. Results indicated a group effect ($\chi^2_{(1, N=25)}$= 9.261; *p*= .002), with the PWS-EG group mainly displaying higher scores than the PWS-CG group. Results also highlighted an effect of the test session ($\chi^2_{(2, N=25)}$= 16.224, *p*< .001) independently of the group.

**REGULATION**

**Table 5- ERC Questionnaire:** Mean (SD) and GLM results for each group and test session

| Group | Pre-test | | Post-test 1 | | Post-test 2 | |
|---|---|---|---|---|---|---|
| GLM Gauss. Dist. | Group effect $\chi^2_{(3, N=75)}$= 31.154, *p*< .001 | | Interaction effect $\chi^2_{(2, N=25)}$=5.988, *p*=.0501 | | Group effect $\chi^2_{(3,N=73)}$=42.507, *p*< .001 | |
| PWS-EG | 3.003 *(0.339)* | Pre-Post1 comp. *z*=-2.74, *p*=.006 | 3.127 *(0.291)* | Post1-2 comp. *ns* | 3.057 *(0.327)* | |
| PWS-CG | 3.069 *(0.240)* | PWS-EG comp. *ns* | 3.036 *(0.231)* | PWS-EG comp. *ns* | 3.072 *(0.215)* | PWS-EG comp. *ns* |
| TD-DA | 3.425 *(0.344)* | *z*= 3.85, *p*<.001 | - | - | - | *z*=3.41, *p*<.001 |
| TD-CA | 3.505 *(0.343)* | *z*=4.58, *p*<.001 | - | - | - | *z*=4.15, *p*<.001 |
